# Clinical efficacy of osimertinib in *EGFR*-mutant non-small cell lung cancer with distant metastasis

**DOI:** 10.1186/s12885-022-09741-8

**Published:** 2022-06-14

**Authors:** Soei Gen, Ichidai Tanaka, Masahiro Morise, Junji Koyama, Yuta Kodama, Akira Matsui, Ayako Miyazawa, Tetsunari Hase, Yoshitaka Hibino, Toshihiko Yokoyama, Tomoki Kimura, Norio Yoshida, Mitsuo Sato, Naozumi Hashimoto

**Affiliations:** 1 Department of Respiratory Medicine, Nagoya University Graduate School of Medicine, 65 Tsurumai-cho, Showa-ku, Nagoya, 466-8550 Japan; 2 Department of Respiratory Medicine, Japanese Red Cross Aichi Medical Center Nagoya Daiichi Hospital, Nagoya, Japan; 3 Department of Respiratory Medicine, Kariya Toyota General Hospital, Kariya, Japan; 4 Department of Respiratory Medicine, Konan Kosei Hospital, Konan, Japan; 5 Department of Respiratory Medicine and Allergy, Tosei General Hospital, Seto, Japan; 6 Division of Host Defense Sciences, Department of Integrated Health Sciences, Nagoya University Graduate School of Medicine, Nagoya, Japan

**Keywords:** Distant metastases, EGFR-TKIs, *EGFR* mutation, Non-small cell lung cancer, Osimertinib

## Abstract

**Background:**

Osimertinib—the third-generation epidermal growth factor receptor (EGFR)-tyrosine kinase inhibitor (TKI)—has been widely used as a first-line treatment for patients with metastatic *EGFR*-mutant non-small cell lung cancer (NSCLC). Osimertinib demonstrated central nervous system activity in patients with brain metastasis; however, its efficacy against other distant metastatic organs, including bone and liver, remains unclear. Therefore, we retrospectively analyzed the clinical efficacy of osimertinib in these patients in comparison to other EGFR-TKIs.

**Methods:**

Clinical data of patients with advanced NSCLC receiving gefitinib/erlotinib (*n* = 183), afatinib (*n* = 55), or osimertinib (*n* = 150) at five medical institutions were retrospectively assessed for progression-free survival (PFS), overall survival (OS), and best overall response rate (ORR).

**Results:**

In univariate and multivariate analyses, most distant metastases, including the brain and bone, were unrelated to the therapeutic efficacy of osimertinib, although liver metastasis and L858R mutation were independently associated with shorter PFS. PFS and OS in patients with liver metastases were significantly shorter than those in patients without liver metastases (PFS: 7.4 vs. 19.7 months, OS: 12.1 months vs. not reached, respectively). Osimertinib provided significantly longer PFS in patients with brain or bone metastasis and exon 19 deletion than the other EGFR-TKIs. The PFS of patients with liver metastases was not significantly different among the three EGFR-TKI groups. Furthermore, the ORR of osimertinib in patients with liver metastases was significantly attenuated, and the effectiveness was similar to 1^st^- or 2^nd^ -generation EGFR-TKIs.

**Conclusion:**

Osimertinib provided better clinical benefits than 1^st^- and 2^nd^-generation EGFR-TKIs for patients with *EGFR*-mutant NSCLC, particularly those with brain or bone metastases and exon 19 deletion; however, its efficacy against liver metastasis was remarkably attenuated. New therapeutic developments for patients with *EGFR*-mutant NSCLC with liver metastases are needed.

**Supplementary Information:**

The online version contains supplementary material available at 10.1186/s12885-022-09741-8.

## Background

Lung cancer is the leading cause of cancer-related mortality worldwide in 2020, and non-small cell lung cancer (NSCLC) is the most common form, accounting for 80% to 85% of all lung cancer diagnoses [1, 2]. The majority of patients with NSCLC are initially diagnosed at an advanced stage with distant metastasis, and the most frequent metastatic sites are the nervous system (20–40%), bone (20–40%), lung (15–25%), and liver (5–20%) [3–5]. Generally, patients with advanced NSCLC with brain, bone, or liver metastases are known to have worse clinical outcomes than patients without metastatic sites [3, 4].

Epidermal growth factor receptor (*EGFR*) mutations, which are one of the targetable driver mutations in NSCLC, are detected in approximately 50% of Asian patients and in approximately 10% of Western patients [6]. In recent decades, EGFR-tyrosine kinase inhibitors (TKIs) have significantly improved the clinical outcomes of patients with *EGFR*-mutant NSCLC, especially in patients with sensitizing EGFR mutations, such as exon 19 deletion and L858R point mutation [7, 8]. These common mutations account for approximately 90% of the total *EGFR* gene alterations [6]. Currently, multiple EGFR-TKIs, including gefitinib, erlotinib, afatinib, dacomitinib, and osimertinib, are established as standard initial treatments for patients with common *EGFR* mutations [7–11]. Among them, the clinical efficacies of 1^st^-generation EGFR-TKIs such as gefitinib and erlotinib have been adequately investigated in patients with distant metastatic sites. Several studies reported that the efficacies for patients with *EGFR*-mutant NSCLC with brain, bone, or liver metastases were limited, and both progression-free survival (PFS) and overall survival (OS) of these patients were remarkably shorter than those of patients without these metastatic sites [12, 13]. The median PFS and OS of patients with brain or bone metastatic sites are around 8.0–9.0 months and 20.0–25.0 months respectively, and the median PFS and OS of patients with liver metastasis are around 6.7 months and 9.2–13.4 months, respectively [12, 13], although the median PFS and OS of patients without these metastatic sites are around 11.0–15.0 months and 17.5–38.0 months, respectively. Additionally, the clinical efficacy of afatinib in patients with brain metastasis is reported to be shorter than that in patients without brain metastasis (10.1 vs 13.9 months) [14]. These results indicate that metastatic sites of *EGFR*-mutant NSCLC are critical factors for EGFR-TKI efficacy and directly affect patient outcomes.

The 3^rd^-generation irreversible EGFR-TKI, Osimertinib, can selectively inhibit both EGFR-TKI sensitizing and T790M resistance mutations, with lower activity against wild-type *EGFR* [15, 16]. In the double-blind phase 3 trial, FLAURA, osimertinib demonstrated significantly longer PFS and OS than the comparator regimens of gefitinib or erlotinib (median PFS: 18.9 vs. 10.2 months; HR, 0.46; *P* < 0.0001; median OS: 38.6 vs. 31.8 months; HR, 0.80; *P* = 0.0460) [17, 18], resulting that osimertinib has become a leading treatment for patients with *EGFR*-mutant NSCLC [17]. In the subgroup analysis of FLAURA, osimertinib demonstrated longer PFS and OS for patients with central nervous system metastases than the regimen of gefitinib or erlotinib (median PFS: 15.2 vs. 9.6 months; HR, 0.47; *P* < 0.0001, median OS: HR, 0.83) [17, 18]. However, the clinical efficacy of osimertinib in patients with NSCLC with other metastatic organs such as bone or liver remains unclear; although, patients with *EGFR*-mutant NSCLC have a higher frequency of bone metastasis than patients with *EGFR*-wild type [5, 19].

To investigate the clinical efficacy of osimertinib in NSCLC with various metastatic organs, we retrospectively analyzed the clinical data of 1^st^ line treatment with osimertinib in patients with common *EGFR*-mutant NSCLC collected from multiple institutions. Furthermore, the efficacies of osimertinib were evaluated in comparison with those of other EGFR-TKIs in a real-world setting.

## Materials and methods

### Study design

This retrospective cohort study was conducted with the approval of the ethical review committee of Nagoya University Hospital (approval number:2018–017) and in accordance with the guidelines of the Declaration of Helsinki [20, 21]. We retrospectively reviewed the medical records of patients from five facilities, including Nagoya University Hospital, Konan Kosei Hospital, Kariya Toyota General Hospital, Tosei General Hospital, and Japanese Red Cross Aichi Medical Center Nagoya Daiichi Hospital. Patients enrolled in this study were selected based on the following eligibility criteria: (1) diagnosed with stage III/IV or recurrent non-squamous NSCLC, as confirmed by histological or cytological examination from January 2015 to December 2020; (2) presented with a positive *EGFR* mutation (exon 19 deletion or L858R point mutation); (3) were receiving 1^st^-generation EGFR-TKI (gefitinib or erlotinib), or 2^nd^-generation EGFR-TKI (afatinib), or 3^rd^-generation EGFR-TKI (osimertinib) for 1^st^ line therapy. We excluded patients with no available data and non-target regions, and the time of data cut-off was August 2021. The clinical information of eligible patients, including age, sex, smoking history, histological subtype, clinical stage, performance status, treatment outcome, metastatic site, and *EGFR* mutation status, were retrospectively obtained from medical records. Clinical stages were assigned according to the eighth edition of the American Joint Committee on Cancer. Objective tumor responses were evaluated according to the Response Evaluation Criteria in Solid Tumors (RECIST) version 1.1 [22].

#### *EGFR* mutation analysis

Genomic DNA was extracted from formalin-fixed paraffin-embedded samples with the QIAamp DNA FFPE Tissue Kit (Qiagen, Hilden, Germany) according to manufacturer’s instructions. Target sequences in exons 19 and 21 were amplified by polymerase chain reaction and the polymerase chain reaction products were then subjected to analysis for *EGFR* mutations by direct Sanger sequencing.

### Statistical analysis

PFS and OS were estimated using the Kaplan–Meier method and were defined as the time from the start of TKI therapy to disease progression or death, whichever was earlier, and data were censored at the last follow-up date. Gehan–Breslow–Wilcoxon and log-rank tests were implemented to analyze the differences in PFS between the patient groups. A Cox regression model was used to estimate hazard ratio (HR) and 95% confidence interval (CI). Categorical data were compared using Fisher’s exact test or the chi-square test. Statistical analyses were performed using JMP software (Version 15) and IBM SPSS Statistics (Version 28), and the differences and correlations were considered statistically significant at *P* < 0.05.

## Results

### Patient’s flowchart and characteristics

We retrospectively reviewed the clinical data of 388 eligible patients with advanced non-squamous NSCLC harboring EGFR mutations treated with EGFR-TKIs as 1^st^-line therapy, which were collected from five medical institutions. The flowchart of patient selection from our medical records is shown in Supplementary Fig. 1, and the enrolled patient characteristics are summarized in Table 1. The median age was 72.0 years (range 26–92), 61.6% were female, and 62.1% did not have a history of smoking. In the majority of patients, the Eastern Cooperative Oncology Group (ECOG) performance status (PS) was 0 (*n* = 230, 59.3%), and the clinical stage was IV (n = 270, 69.6%). Exon 19 deletions were observed in 188 patients (48.6%), and L858R point mutations were observed in 199 patients (51.4%). The number of patients with other metastatic organs included 92 (23.7%) for the contralateral lung, 160 (41.2%) for the bone, 118 (30.4%) for the brain, 34 (8.8%) for the liver, and 26 (6.7%) for the adrenal, which are consistent with the frequencies of *EGFR*-mutant NSCLC metastases in previous reports [3–5]. In this cohort, 183, 55, and 150 patients were treated with gefitinib, erlotinib, afatinib, and osimertinib, respectively. There were no statistically significant differences in sex, smoking status, performance status (PS), stage, and metastatic organs between gefitinib/erlotinib, afatinib, and osimertinib groups. The proportion of exon 19 deletion mutations in the afatinib group was higher than that in the other TKI groups (*P* < 0.0001), and the patients treated with afatinib were significantly younger than those in the other groups (*P* < 0.0001).

**Table 1:** Clinical characteristics of 388 patients with NSCLC

		**EGFR-TKIs n (%)**			
**Characteristic**	**Total**	**Gefitinib/** **Erlotinib**	**Afatinib**	**Osimertinib**	***P***^‡^
	388	183	55	150	
**Median Age (Range)**		73 (26–87)	65 (32–79)	72 (44–92)	< 0.0001
**Gender**					
Male	149	64 (35.0)	29 (52.7)	56 (37.3)	0.0563
Female	239	119 (65.0)	26 (47.3)	94 (62.7)	
**Smoking status**^a^					
Never	236	117 (64.6)	26 (49.0)	93 (63.7)	0.0868
Former	104	49 (27.1)	16 (30.2)	39 (26.7)	
Current	40	15 (8.3)	11 (20.8)	14 (9.6)	
**PS**					
0	230	110 (60.1)	33 (60.0)	87 (58.0)	0.7914
1	99	42 (23.0)	15 (27.3)	42 (28.0)	
≧2	59	31 (16.9)	7 (12.7)	21 (14.0)	
**Stage**					
III	15	9 (4.9)	0 (0.0)	6 (4.0)	0.8257
IV	270	125 (68.3)	40 (72.7)	105 (70.0)	
Recurrence	103	49 (26.8)	15 (27.3)	39 (26.0)	
**Mutation status**^b^					
Exon 19 deletion	188	76 (41.8)	44 (80.0)	68 (45.3)	< 0.0001
L858R	199	106 (58.2)	11 (20.0)	82 (54.7)	
**Metastasis**					
Pleura	144	72 (39.3)	16 (29.1)	56 (37.3)	0.3848
Contralateral lung	92	40 (21.9)	12 (21.8)	40 (26.7)	0.5541
Bone	160	68 (37.2)	29 (52.7)	63 (42.0)	0.1171
Brain	118	50 (27.3)	20 (36.4)	48 (32.0)	0.3820
Liver	34	14 (7.7)	3 (5.5)	17 (11.3)	0.3205
Adrenal	26	13 (7.1)	6 (10.9)	7 (4.7)	0.2728

*PS* performance status

^‡^*P* values were calculated by t-test, Fisher’s exact test, or Chi-square test

^a^Information was not available for 8 cases

^b^Information was not available whether it was exon 19del or L858R for 1 case

### Liver metastasis—an independent prognostic factor

**Fig. 1:**
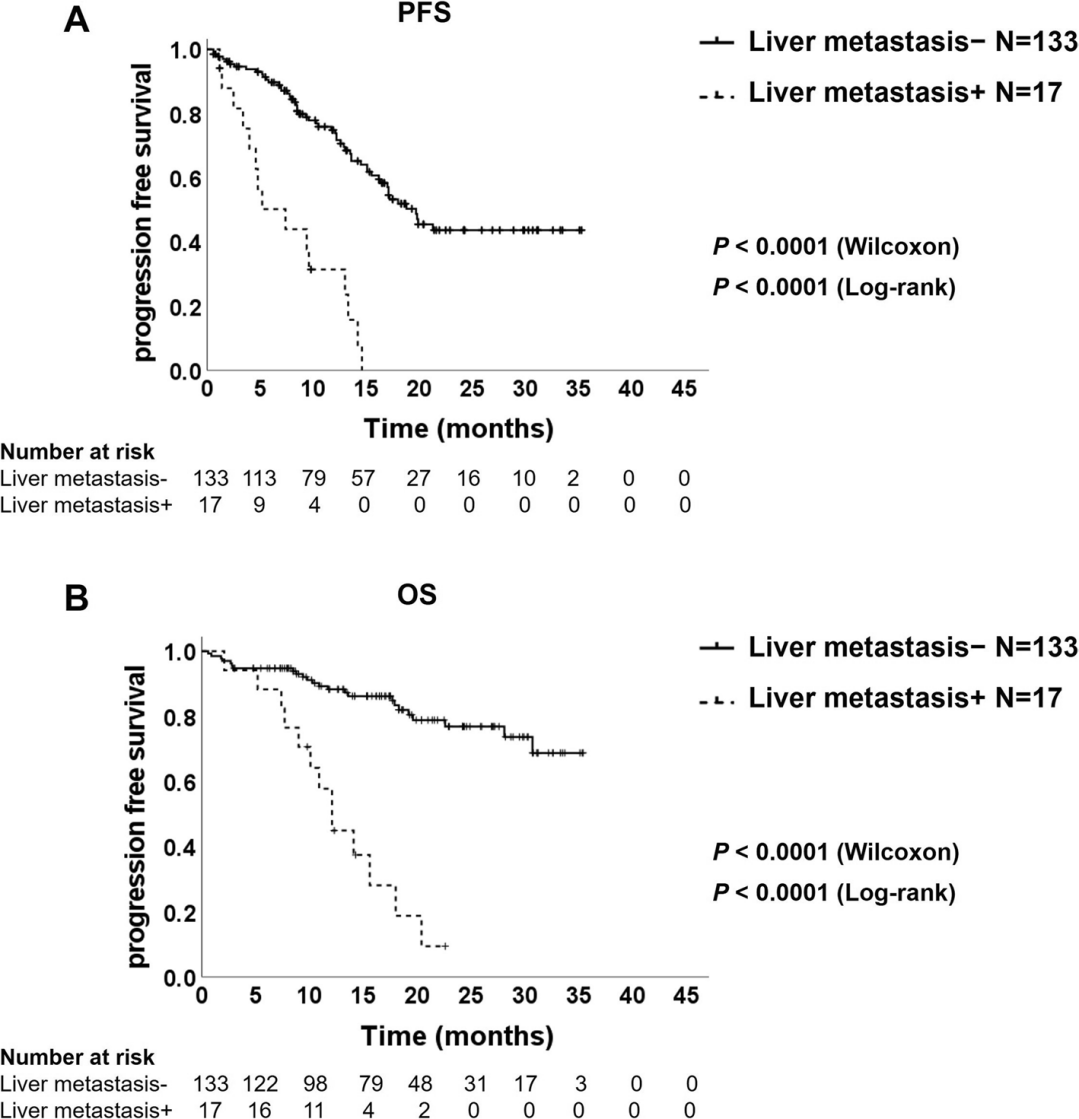
Kaplan–Meier plot of progression-free survival (**A**) and overall survival (**B**) in the patients treated with osimertinib with and without liver metastases

First, we performed univariate and multivariate analyses of PFS in each of the three groups. In addition to brain, bone, and liver metastases, the characteristic variables with *P* < 0.15 in univariate analysis of the osimertinib group were used in the multivariate analysis. In the osimertinib group, male sex, poor PS, L858R mutation, and liver metastasis were independently associated with shorter PFS (male: HR, 2.06; 95% CI, 1.25–3.38; *P* = 0.0045, PS1: HR, 2.70; 95% CI, 1.47–4.94; *P* = 0.0013, PS≧2: HR, 2.43; 95% CI, 1.16–5.11; *P* = 0.0193, L858R mutation: HR, 2.03; 95% CI, 1.17–3.53; *P* = 0.0120; liver metastasis: HR, 6.20; 95% CI, 2.87–13.38; *P* < 0.0001, Table 2). Among them, the PFS in patients with liver metastases was significantly shorter than that in patients without liver metastases (7.4 vs. 19.7 months; Wilcoxon *P* < 0.0001 and log-rank *P* < 0.0001; Fig. 1A), while the other distant metastatic sites were not associated with shorter PFS (Table 2). In addition, the OS in patients with liver metastases was remarkably shorter than in patients without liver metastases (12.1 months vs not reached; Wilcoxon *P* < 0.0001 and log-rank *P* < 0.0001; Fig. 1B). In the gefitinib/erlotinib group, poor PS (PS ≥ 2) was an independent poor prognostic factor (HR, 1.83; 95% CI, 1.14–2.94; *P* = 0.0124), although brain, bone, and liver metastases were not associated with shorter PFS (brain: HR, 1.15; 95% CI, 0.79–1.66; *P* = 0.4663; Bone: HR, 1.17; 95% CI, 0.83–1.66; *P* = 0.3712; Liver: HR, 1.44; 95% CI, 0.81–2.57; *P* = 0.2108, Supplementary Table 1A). Similarly, in the afatinib group, metastasis sites were not statistically associated with PFS (brain, HR, 1.42; 95% CI, 0.74–2.73; *P* = 0.2950; Bone, HR, 1.19; 95% CI, 0.59–2.42; *P* = 0.6258; Liver, HR, 2.09; 95% CI, 0.50–8.71; *P* = 0.3132; Supplementary Table 1B).

**Table 2:** univariate and multivariate analysis of PFS in Osimertinib group

	**Univariate analysis**			**Multivariable analysis**		
**Variable**	HR	95% Cl	*P*	HR	95% Cl	*P*
**Gender**						
Female	Reference			Reference		
Male	1.58	0.98–2.54	0.0592	2.06	1.25–3.38	0.0045
**Age**						
≦65 years	Reference			Reference		
> 65 years	1.58	0.85–2.95	0.1484	1.14	0.57–2.28	0.7027
**Smoking status**						
Never smoker	Reference					
Former smoker	1.41	0.83–2.41	0.2024			
Current smoker	1.08	0.46–2.57	0.8450			
**Stage**						
Recurrence	Reference					
III	0.82	0.19–3.57	0.7959			
IV	1.14	0.66–1.97	0.6447			
**PS**						
0	Reference			Reference		
1	2.61	1.53–4.46	0.0004	2.70	1.47–4.94	0.0013
≧2	2.73	1.45–5.12	0.0018	2.43	1.16–5.11	0.0193
**Mutation**						
Exon 19 deletion	Reference			Reference		
L858R	2.22	1.32–3.71	0.0025	2.03	1.17–3.53	0.0120
**Bone metastasis**						
No	Reference			Reference		
Yes	1.19	0.74–1.91	0.4751	0.62	0.35–1.09	0.0956
**Brain metastasis**						
No	Reference			Reference		
Yes	1.44	0.89–2.34	0.1353	1.27	0.73–2.22	0.3924
**Liver metastasis**						
No	Reference			Reference		
Yes	5.14	2.81–9.41	< 0.0001	6.20	2.87–13.38	< 0.0001
**Pleura metastasis**						
No	Reference					
Yes	1.36	0.84–2.19	0.2148			
**Lung metastasis**						
No	Reference					
Yes	1.33	0.79–2.23	0.2786			
**Adrenal metastasis**						
No	Reference			Reference		
Yes	1.98	0.80–4.95	0.1420	0.45	0.15–1.36	0.1581

*PS* performance status

### Improved brain/bone metastases prognosis

**Fig. 2:**
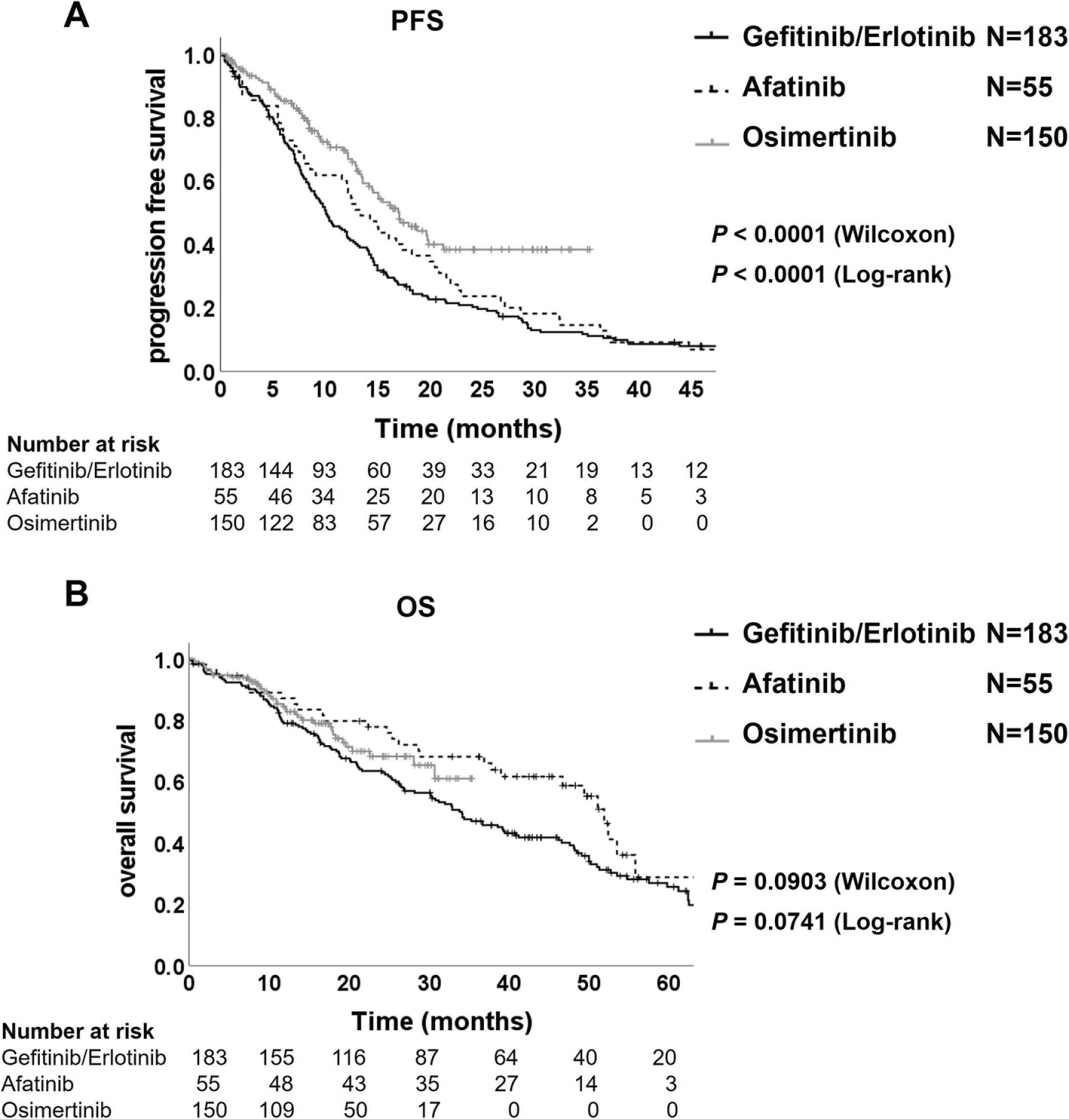
Kaplan–Meier plot of progression-free survival (**A**) and overall survival (**B**) in the patients treated by gefitinib/erlotinib, afatinib or osimertinib

In comparison with the clinical efficacy of EGFR-TKIs, the PFS in the patients treated by osimertinib was statistically significantly longer than in the patients treated by gefitinib/erlotinib or afatinib (17.1 vs. 10.1 months; Wilcoxon *P* < 0.0001, log-rank *P* < 0.0001; HR, 0.52; 95% CI, 0.39–0.69; and 17.1 vs. 13.4 months; Wilcoxon *P* = 0.0541, log-rank *P* = 0.0250; HR, 0.65; 95% CI, 0.45–0.95; Fig. 2A). Furthermore, the patients of the osimertinib treatment group showed longer OS than the patients of the gefitinib/erlotinib treatment group, although not statistically significant (not reached vs. 34.1 months; Wilcoxon *P* = 0.2150 and log-rank *P* = 0.1818; HR, 0.77; 95% CI, 0.52–1.13; Fig. 2B), and there were no significant differences of OS in between osimertinib and afatinib groups (Fig. 2B).

**Fig. 3:**
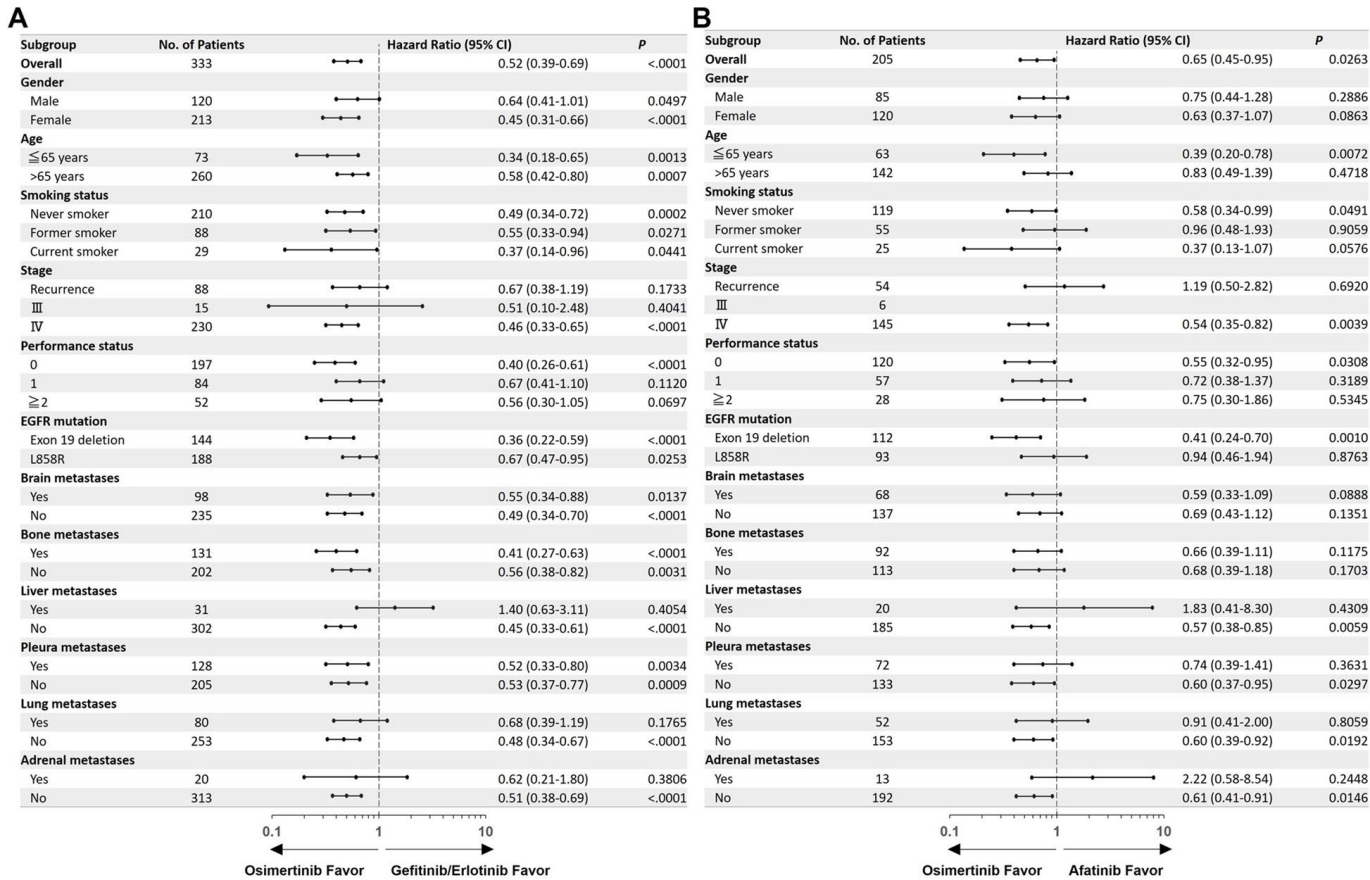
Subgroup analyses of progression-free survival. Osimertinib compared with gefitinib/erlotinib (**A**) or afatinib (**B**). A hazard ratio of less than one implies a lower risk of disease progression or death with osimertinib than with other EGFR-TKIs. Smoking status were not available for 2 patients in the gefitinib/erlotinib group, 2 patients in the afatinib group and 4 patients in the osimertinib group. Information was not available whether it was exon 19 deletion or L858R mutation for 1 patient in gefitinib/erlotinib group. There were no patient with stage III in afatinib group. CI, confidence interval

Patient characteristics with brain, bone, and liver metastases are summarized in Supplementary Table 2A, 2B, and 2C, respectively. The number of patients with brain metastases treated with stereotactic radiosurgery was significantly lower in the osimertinib group than in the other TKI groups (*P* = 0.0239; Supplementary Table 2A). The number of bone metastatic sites, treatment with bone-modifying agents, or radiation therapy was not significantly different between the gefitinib/erlotinib, afatinib, and osimertinib groups (Supplementary Table 2B), and the number of liver metastatic sites were also not significantly different between the gefitinib/erlotinib, afatinib, and osimertinib groups (Supplementary Table 2C). In comparison with gefitinib/erlotinib, the forest plots of PFS showed that osimertinib was associated with a significant survival benefit in brain metastases (HR, 0.55; 95% CI, 0.34–0.88; *P* = 0.0137), bone (HR, 0.41; 95% CI, 0.27–0.63; *P* < 0.0001) and pleura (HR, 0.52; 95% CI, 0.33–0.80; *P* = 0.0034) (Fig. 3A). On the contrary, the largest numerical differences in the hazard ratio between osimertinib and the gefitinib/erlotinib group were observed in the patients with and without liver metastases (HR, 1.40; 95% CI, 0.63–3.11; *P* = 0.4054); however, there were no statistical differences between osimertinib and gefitinib/erlotinib in the subgroup with contralateral lung metastases and adrenal metastases (Fig. 3A). The second largest numerical difference was observed between exon 19 deletion and L858R mutations (Fig. 3A). Similar to previous studies [17], osimertinib was associated with a significant survival benefit in the exon 19 deletion subgroup (HR, 0.36; 95% CI, 0.22–0.59; *P* < 0.0001) compared to the L858R subgroup (HR, 0.67; 95% CI, 0.47–0.95; *P* = 0.0253).

Similarly, in comparison with afatinib, osimertinib was associated with better survival benefit in the brain and bone metastases subgroup, although the difference was not statistically significant (brain: HR, 0.59; 95% CI, 0.33–1.09; *P* = 0.0888; Bone: HR, 0.66; 95% CI, 0.39–1.11; *P* = 0.1175) (Fig. 3B). In contrast, the largest numerical differences were observed between patients with and without liver or adrenal metastases, indicating that the clinical benefits of osimertinib are weak in patients with liver metastases and adrenal metastases (liver: HR, 1.83; 95% CI, 0.41–8.30; *P* = 0.4309, adrenal; HR, 2.22; 95% CI, 0.58–8.54; *P* = 0.2448; Fig. 3B). Additionally, large numerical differences were observed between the exon 19 deletion and L858R mutation, indicating that osimertinib was associated with a significant survival benefit in the exon 19 deletion subgroup (HR, 0.41; 95% CI, 0.24–0.70; *P* = 0.0010), but not in the L858R subgroup (HR, 0.94; 95% CI, 0.46–1.94; *P* = 0.8763) (Fig. 3B).

**Fig. 4:**
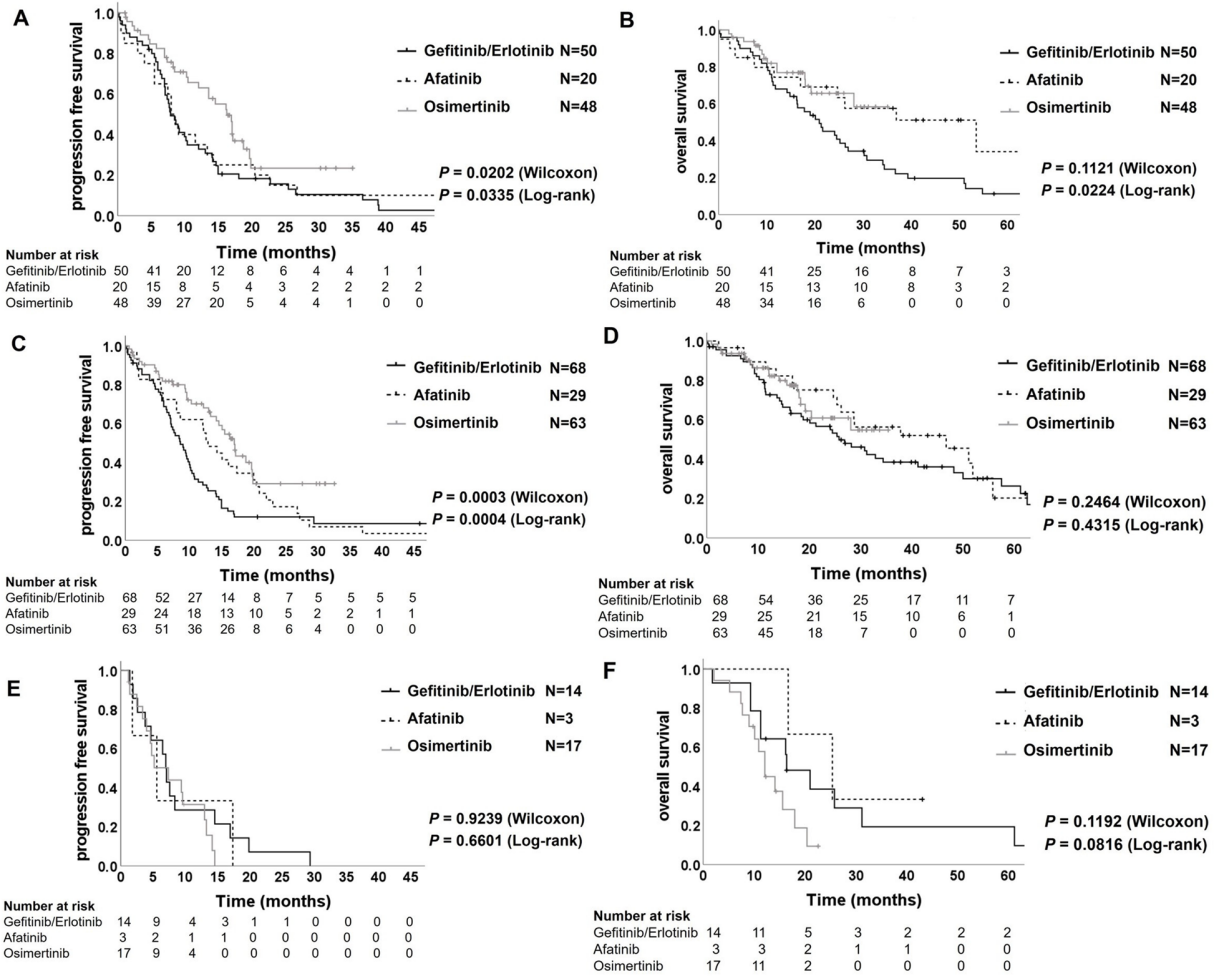
Kaplan–Meier plot of progression-free survival and of overall survival in the patients with brain metastases (**A**) and (**B**), respectively, in the patients with bone metastases (**C**) and (**D**), respectively, and in the patients with liver metastases (**E**) and (**F**), respectively

Subsequently, we analyzed the clinical efficacy of EGFR-TKIs in patients with distant organ metastasis using the Kaplan–Meier estimator. The PFS of the patients with brain metastasis treated by osimertinib was significantly longer than those of the patients of the gefitinib/erlotinib group (16.3 vs. 7.9 months; Wilcoxon *P* = 0.0075 and log-rank *P* = 0.0120), and the afatinib group (16.3 vs. 8.3 months; Wilcoxon *P* = 0.0347 and log-rank *P* = 0.0845) (Fig. 4A). Furthermore, the OS in the patients with brain metastasis treated by osimertinib was significantly longer than in the patients treated by gefitinib/erlotinib (not reached vs. 20.9 months; Wilcoxon *P* = 0.0725 and log-rank *P* = 0.0326), while there was not significantly difference between the osimertinib and the afatinib group (not reached vs. 53.5 months; Wilcoxon *P* = 0.6219 and log-rank *P* = 0.8118) (Fig. 4B). These results indicate that the clinical efficacy of osimertinib for patients with *EGFR*-mutant NSCLC with brain metastases is equal to or greater than that of the other EGFR-TKIs, and our analyzed data were consistent with the previous reports of the FLAURA trial [17, 18]. Furthermore, in patients with bone metastasis, the PFS of the osimertinib group was significantly longer than that of the gefitinib/erlotinib group (17.0 vs. 8.6 months; Wilcoxon *P* < 0.0001 and log-rank *P* < 0.0001; Fig. 4C) and showed a better trend compared with those of the patients in the afatinib group (17.0 vs. 12.9 months; Wilcoxon *P* = 0.1884 and log-rank *P* = 0.1144; Fig. 4C), although the OS demonstrated no significant differences among the three EGFR-TKI groups (Fig. 4D). However, the PFS in the patients with liver metastasis of osimertinib group showed no superiority to the patients of the gefitinib/erlotinib group (7.4 vs. 7.1 months; Wilcoxon *P* = 0.7406 and log-rank *P* = 0.3997; Fig. 4E) and the afatinib group (7.4 vs. 5.6 months; Wilcoxon *P* = 0.8674 and log-rank *P* = 0.4247; Fig. 4E). Similar to the PFS analyses, the OS of patients with liver metastasis treated with osimertinib showed no significant difference compared to the patients treated with 1^st^- or 2^nd^-generation EGFR-TKIs (Fig. 4F). However, the PFS in patients without liver metastasis was significantly better in the osimertinib group than in the other EGFR-TKI groups (Supplementary Fig. 2A). The OS in the patients without liver metastasis in the osimertinib group was significantly better than that of the patients in the gefitinib/erlotinib group, while there was no significant difference between the osimertinib and afatinib groups (Supplementary Fig. 2B). These results indicate that liver metastasis critically affects the clinical efficacy of osimertinib in patients with *EGFR*-mutant NSCLC.

### Attenuated clinical efficacy of osimertinib

**Fig. 5:**
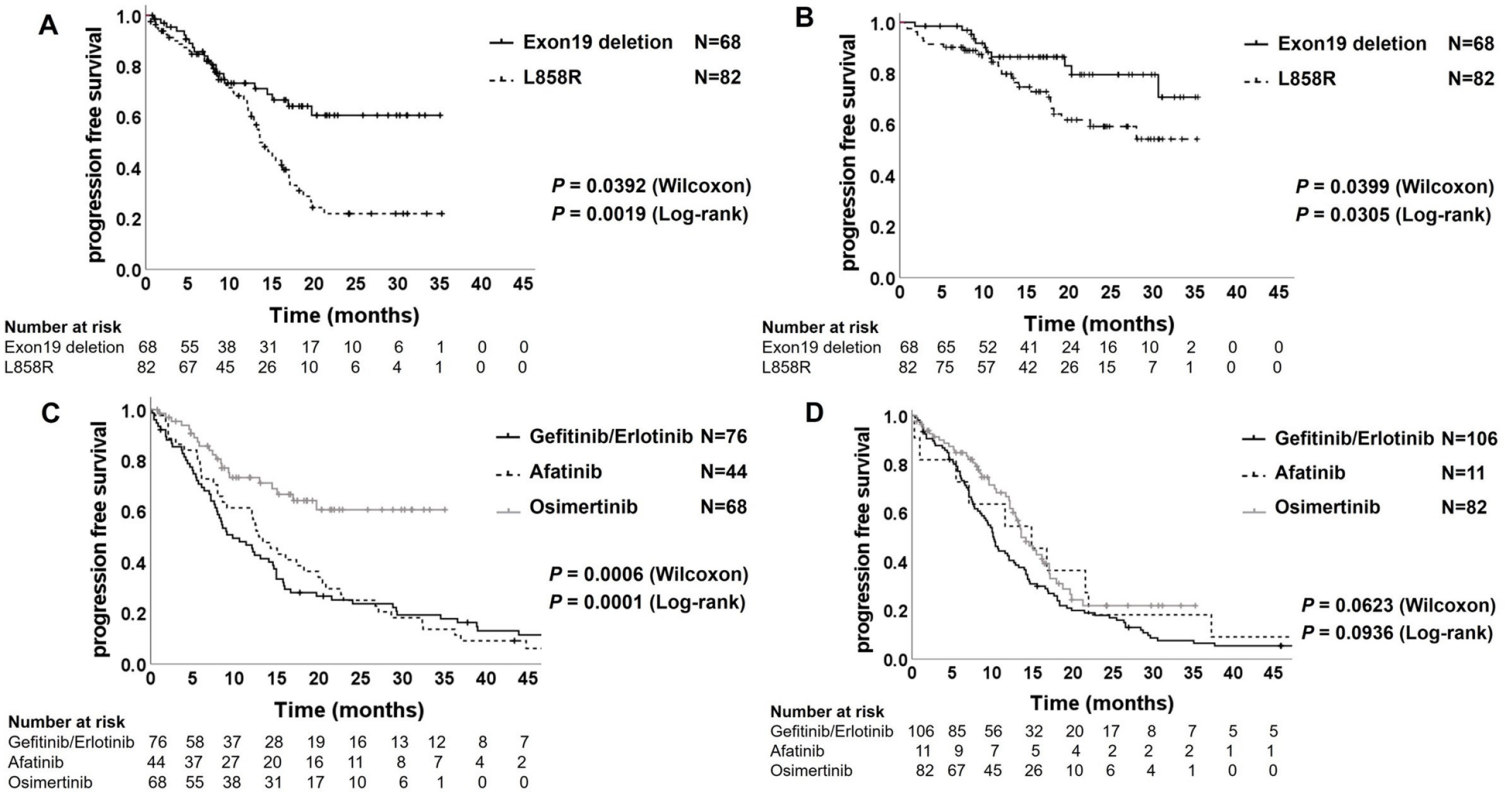
Kaplan–Meier plot of progression-free survival (**A**) and overall survival (**B**) in the patients treated by osimertinib with exon 19 deletion or L858R mutation. Kaplan–Meier plot of progression-free survival in the patients with exon 19 deletion mutation (**C**) and in the patients with L858R mutation (**D**) treated by gefitinib/erlotinib, afatinib or osimertinib

In patients treated with osimertinib, the PFS and OS of patients with exon 19 deletions were significantly longer than those of patients with the L858R mutation (Fig. 5A and Fig. 5B). As expected, the PFS in the patients with exon 19 deletion mutation in the osimertinib group was significantly better than that in the patients in the gefitinib/erlotinib group and the afatinib group (not reached vs. 9.8 months; Wilcoxon *P* = 0.0001 and log-rank *P* < 0.0001, and not reached vs. 13.2 months; Wilcoxon *P* = 0.0085 and log-rank *P* = 0.0007; Fig. 5C). However, the PFS of the patients with L858R mutation did not show a significant difference between the osimertinib group and the afatinib group (13.6 vs. 14.9 months; Wilcoxon *P* = 0.6038 and log-rank *P* = 0.8761; Fig. 5C); though, osimertinib showed superior median PFS to gefitinib/erlotinib (13.6 vs. 10.2 months; Wilcoxon *P* = 0.0176 and log-rank *P* = 0.0239; Fig. 5D). On the other hand, the OS of patients with both exon 19 deletion and L858 mutation in the osimertinib group were not significantly different from those in the other EGFR-TKI groups (Supplementary Fig. 3A and 3B).

### Limited response rate of osimertinib

To assess the response rate to osimertinib, we compared the ORRs in patients treated with each EGFR-TKI. The ORRs were 68.2% (116/170) in the gefitinib/erlotinib group, 59.6% (31/52) in the afatinib group, and 73.9% (99/134) in the osimertinib group, indicating that osimertinib demonstrated a slightly better response rate than the other EGFR-TKIs (Table 3). However, the response rate in patients with liver metastasis treated with osimertinib was remarkably reduced, and the ORRs were 53.3% (8/15), while those of the gefitinib/erlotinib and afatinib groups were 57.1% (8/14) and 66.7% (2/3), respectively (Table 4 and Supplementary Fig. 4A). Consistent with the results of PFS and OS, the clinical response to osimertinib in patients with liver metastases was significantly attenuated, and the effectiveness was similar to 1^st^- or 2^nd^-generation EGFR-TKIs.

**Table 3:** Best response in each treatment population

	**Gefitinib/Erlotinib** **n (%)**	**Afatinib** **n (%)**	**Osimertinib** **n (%)**
**Total**^a^	170	52	134
**Best Response**			
CR	17 (10.0)	5 (9.6)	5 (3.7)
PR	99 (58.2)	26 (50.0)	94 (70.1)
SD	38 (22.4)	15 (28.8)	26 (19.4)
PD	16 (9.4)	6 (11.5)	9 (6.7)
**ORR**	116 (68.2)	31 (59.6)	99 (73.9)
**DCR**	154 (90.6)	46 (88.5)	125 (93.3)

**Table 4:** Best response in patients with/without liver metastasis

	**Gefitinib/Erlotinib**		**Afatinib**		**Osimertinib**	
	**Liver metastasis + n (%)**	**Liver metastasis-** **n (%)**	**Liver metastasis + n (%)**	**Liver metastasis-** **n (%)**	**Liver metastasis + n (%)**	**Liver metastasis-** **n (%)**
**Total **^**a**^	14	156	3	49	15	119
**Best Response**						
CR	0 (0.0)	17 (10.9)	0 (0.0)	5 (10.2)	0 (0.0)	5 (4.2)
PR	8 (57.1)	91 (58.3)	2 (66.7)	24 (48.9)	8 (53.3)	86 (72.3)
SD	2 (14.3)	36 (23.1)	0 (0.0)	15 (30.6)	4 (26.7)	22 (18.5)
PD	4 (28.6)	12 (7.7)	1 (33.3)	5 (10.2)	3 (20.0)	6 (5.0)
**ORR**	8 (57.1)	108 (69.2)	2 (66.7)	29 (59.2)	8 (53.3)	91 (76.5)
**DCR**	10 (71.4)	144 (92.3)	2 (66.7)	44 (89.8)	12 (80.0)	113 (95.0)

In addition, in patients with exon 19 deletion mutations, the ORR of the osimertinib group was 76.2% (48/63), whereas that of the gefitinib/erlotinib and afatinib groups was 66.7% (46/69) and 57.1% (24/42), respectively (Table 5 and Supplementary Fig. 4B). However, in patients with L858R mutations, the ORRs of the osimertinib group were 71.8% (51/71), whereas those of the gefitinib/erlotinib and afatinib groups were 70.0% (70/100) and 70.0% (7/10), respectively (Table 5 and Supplementary Fig. 4B). In addition to the analyses of PFS and OS, the ORRs in patients with exon 19 deletions treated with osimertinib were superior to those in patients with other EGFR-TKIs; while those in patients with L858R did not show remarkable differences among the three groups.

**Table 5:** Best response in patients by *EGFR* mutations

	**Gefitinib/Erlotinib**		**Afatinib**		**Osimertinib**	
	**Exon 19 del** **n (%)**	**L858R** **n (%)**	**Exon 19 del** **n (%)**	**L858R** **n (%)**	**Exon 19 del** **n (%)**	**L858R** **n (%)**
**Total**^a^	69	100	42	10	63	71
**Best Response**						
CR	11 (15.9)	6 (6.0)	3 (7.1)	2 (20.0)	3 (4.8)	2 (2.8)
PR	35 (50.7)	64 (64.0)	21 (50.0)	5 (50.0)	45 (71.4)	49 (69.0)
SD	16 (23.2)	22 (22.0)	14 (33.3)	1 (10.0)	9 (14.3)	17 (24.20)
PD	7 (10.1)	8 (8.0)	4 (9.5)	2 (20.0)	6 (9.5)	3 (4.2)
**ORR**	46 (66.7)	70 (70.0)	24 (57.1)	7 (70.0)	48 (76.2)	51 (71.8)
**DCR**	62 (89.9)	92 (92.0)	38 (90.5)	8 (80.0)	57 (90.5)	68 (95.8)

*CR* complete response, *PR* partial response, *SD* stable disease, *PD* progressive disease, *ORR* objective response rate, *DCR* disease control rate, *exon 19 del* exon 19 deletion

^a^There were 13 patients in the gefitinib/erlotinib group, 3 cases in the afatinib group, and 16 cases in the osimertinib group could not be evaluated

## Discussion

To our knowledge, this is the first report of the efficacy of osimertinib in comparison with other EGFR-TKIs in a real-world setting. Overall, osimertinib provided better clinical benefits to patients with EGFR-mutated NSCLC, particularly those with brain and bone metastases, and exon 19 deletion than 1^st^- and 2^nd^-generation EGFR-TKIs, while the efficacy was attenuated in patients with liver metastases and L858R mutation.

In this study, PFS, OS, and ORRs of patients treated with osimertinib or 1^st^-generation EGFR-TKIs were similar to the results of the FLAURA trial, and the HR of PFS was also very close to the trial result. Furthermore, our study demonstrated good clinical efficacy of osimertinib for patients with brain metastasis and exon 19 deletion, and these results are consistent with a previous report [17, 18]. Among various distant metastatic sites, this study is the first to demonstrate the clinical efficacy of osimertinib in patients with bone metastases who had a poor prognosis in the case of 1^st^-generation EGFR-TKIs treatments [12]. In vivo analysis showed that osimertinib effectively regressed tumors in an *EGFR*-mutant bone metastatic model [23], indicating that the tissue penetration rate of osimertinib can be greater than that of other EGFR-TKIs. However, our study showed remarkably lower efficacy of osimertinib as well as other EGFR-TKIs in patients with liver metastasis. Moreover, the same trend was observed in another retrospective study [24], suggesting that the clinical efficacy of any EGFR-TKI monotherapy is limited to NSCLC with liver metastasis. In the tumor microenvironment of the liver metastatic site, the expression level of vascular endothelial growth factor (VEGF) is increased in comparison with other metastatic sites [25]. VEGF induces not only tumor angiogenesis, but also promotes the proliferation of *EGFR*-mutant cancer cells and affects the immune suppressive network [26–28]. In fact, combination therapy with erlotinib plus anti-VEGF receptor antibody, ramucirumab, shows better HR of PFS in patients with liver metastases than erlotinib monotherapy [29]. Furthermore, insulin-like growth factor 1 (IGF-1), which is a ligand of the IGF-1 receptor (IGF-1R), is abundantly expressed in the microenvironment of the liver metastatic site, and the signaling from IGF-1R promotes the tolerance to osimertinib in *EGFR*-mutant tumors [30]. These liver-specific tumor microenvironments may reduce the clinical efficacy of osimertinib in patients with liver metastasis.

Similar to previous reports [17, 18], our study also demonstrated that the clinical efficacy of osimertinib was limited in patients with the L858R mutation, although osimertinib showed longer median PFS than 1^st^-generation TKIs. Osimertinib was developed for its activity against the T790M mutation, which is a secondary point mutation in EGFR and is the most common resistance mechanism to 1^st^- and 2^nd^-generation EGFR-TKIs [31, 32]. A recent study showed that the prevalence of the T790M mutation was significantly higher in patients with exon 19 deletions than in those with the L858R mutation (50.4% versus 36.5%) [33], indicating that osimertinib is more beneficial in patients with exon 19 deletions. Our data showed that the PFS of patients with exon 19 deletion in the osimertinib group was significantly longer than that in the afatinib group; however, there was no significant difference in PFS in patients with the L858R mutation between osimertinib and afatinib. To investigate the clinical benefits of osimertinib compared with afatinib in patients with the L858R mutation, further studies are needed.

Although this study showed the superiority of osimertinib in PFS over afatinib, there were no remarkable differences in OS between the two EGFR-TKIs. In our cohort, the afatinib group included a high proportion of patients with exon19 deletion mutation and relatively younger patients. Furthermore, after the failure of 1^st^-line gefitinib/erlotinib or afatinib, osimertinib was used for 33.3% patients of the gefitinib/erlotinib group and 27.3% patients of the afatinib group as 2^nd^-line treatment (Supplementary Table 3), while the osimertinib group did not include other EGFR-TKIs therapy after 2^nd^-line treatments. Most T790M-positive patients were treated with osimertinib as 2^nd^-line treatment after 1^st^-line afatinib (Supplementary Table 3). These factors may have affected the good OS of the afatinib group. Furthermore, this study has some limitations, including retrospectively analyzed results and a limited population, especially the number of patients treated with afatinib. In addition, OS analysis required a longer follow-up period, particularly for patients treated with osimertinib. To clearly show the clinical benefits of osimertinib in comparison with 2^nd^-generation EGFR-TKIs, large-scale clinical trials are necessary.

## Conclusion

The clinical efficacy of osimertinib was better in most cases of *EGFR*-mutant NSCLC than in those of 1^st^- or 2^nd^-generation EGFR-TKIs. Osimertinib provided significantly better clinical outcomes in patients with exon 19 deletions and brain or bone metastases and demonstrated significantly shorter PFS in patients with liver metastasis than in patients with other metastatic organs. The clinical efficacy did not show superiority to 1^st^- and 2^nd^-generation EGFR-TKIs. These results indicate that new therapeutic strategies for patients with *EGFR*-mutant NSCLC with liver metastasis are urgently needed.

## Supplementary Information

**Additional file 1: Supplementary Table 1A.** univariate and multivariateanalysis of PFS in Gefitinib/Erlotinib group. **Supplementary Table 1B.** univariate and multivariate analysis of PFS in Afatinib group. **Supplementary Table 2A.** Clinicalcharacteristics of 118 patients with brain metastases. **Supplementary Table 2B.** Clinical characteristics of 160 patientswith bone metastases. **Supplementary Table2C.** Clinical characteristics of 34 patients with liver metastases. **Supplementary Table 3.** Second-linetreatments afterGefitinib/Erlotinib or Afatinib failure. **Supplementary Figure 1**.Flowchartof patient selection in this cohort. **Supplementary Figure 2.** Kaplan–Meier plot ofprogression-free survival (A) and overall survival (B) in patients withoutliver metastases. **Supplementary Figure 3.** Kaplan–Meier plot ofoverall survival in patients with exon 19 deletion mutation (A) in patientswith the L858R mutation (B). **Supplementary Figure 4.** Objective response ratein three EGFR-TKI groups with or without liver metastasis (A) and exon 19deletion or L858R (B). Tumor responses were assessed by the investigatorsaccording to the Response Evaluation Criteria in Solid Tumors (RECIST) version1.1. *P *values were calculated using the chi-square test. Thirteen casesin the gefitinib/erlotinib group, three in the afatinib group, and 16 in theosimertinib group could not be evaluated.

**Additional file 2.** Supplementary data.

## Data Availability

The datasets used and analysed during the current study are available from the corresponding author on reasonable request.

## Abbreviations

EGFR: Epidermal Growth Factor Receptor
NSCLC: Non-Small Cell Lung Cancer
ORR: Overall Response Rate
OS: Overall Survival
PFS: Progression-Free Survival
TKI: Tyrosine Kinase Inhibitor
PS: Performance Status
