# Supplementary material for: Clinical efficacy of osimertinib in EGFR-mutant non-small cell lung cancer with distant metastasis
Source: BMC Cancer. 2022 Jun 14;22:654. doi: 10.1186/s12885-022-09741-8 (PMC9195197; doi:10.1186/s12885-022-09741-8)
Supplement: Supplementary file 1 — Additional file 1: Supplementary Table 1A. univariate and multivariateanalysis of PFS in Gefitinib/Erlotinib group. Supplementary Table 1B. univariate and multivariate analysis of PFS in Afatinib group. Supplementary Table 2A. Clinicalcharacteristics of 118 patients with brain metastases. Supplementary Table 2B. Clinical characteristics of 160 patientswith bone metastases. Supplementary Table2C. Clinical characteristics of 34 patients with liver metastases. Supplementary Table 3. Second-linetreatments afterGefitinib/Erlotinib or Afatinib failure. Supplementary Figure 1.Flowchartof patient selection in this cohort. Supplementary Figure 2. Kaplan–Meier plot ofprogression-free survival (A) and overall survival (B) in patients withoutliver metastases. Supplementary Figure 3. Kaplan–Meier plot ofoverall survival in patients with exon 19 deletion mutation (A) in patientswith the L858R mutation (B). Supplementary Figure 4. Objective response ratein three EGFR-TKI groups with or without liver metastasis (A) and exon 19deletion or L858R (B). Tumor responses were assessed by the investigatorsaccording to the Response Evaluation Criteria in Solid Tumors (RECIST) version1.1. P values were calculated using the chi-square test. Thirteen casesin the gefitinib/erlotinib group, three in the afatinib group, and 16 in theosimertinib group could not be evaluated. [file 12885_2022_9741_MOESM1_ESM.docx]

**Supplementary Information**

**Clinical efficacy of osimertinib in *EGFR*-mutant non-small cell lung cancer with distant metastasis**

Soei Gen^1^, Ichidai Tanaka^1^*, Masahiro Morise^1^, Junji Koyama^1^, Yuta Kodama^2^, Akira Matsui^3^,　Ayako Miyazawa^4^, Tetsunari Hase^1^, Yoshitaka Hibino^4^, Toshihiko Yokoyama^2^, Tomoki Kimura^5^, Norio Yoshida^3^, Mitsuo Sato^6^, Naozumi Hashimoto^1^

**Supplementary Table 1A. univariate and multivariate analysis of PFS in Gefitinib/Erlotinib group**

|  | **Univariate analysis** | | | **Multivariable analysis** | | |
| --- | --- | --- | --- | --- | --- | --- |
| **Variable** | HR | 95% Cl | *P-value* | HR | 95% Cl | *P-value* |
| **Gender** |  | | | | | |
| Female | Reference | | | Reference | | |
| Male | 1.09 | 0.80-1.51 | 0.5714 | 1.13 | 0.81-1.60 | 0.4507 |
| **Age** |  | | | | | |
| ≦65 years | Reference | | | Reference | | |
| >65 years | 0.99 | 0.68-1.42 | 0.9593 | 1.05 | 0.72-1.52 | 0.8043 |
| **Smoking status** |  | | | | | |
| Never smoker | Reference | | |  | | |
| Former smoker | 1.38 | 0.97-1.96 | 0.0721 |  |  |  |
| Current smoker | 1.55 | 0.88-2.72 | 0.1286 |  |  |  |
| **Stage** |  | | | | | |
| Recurrence | Reference | | |  | | |
| Ⅲ | 0.82 | 0.37-1.82 | 0.6201 |  |  |  |
| Ⅳ | 1.53 | 1.07-2.18 | 0.0188 |  |  |  |
| **PS** |  | | | | | |
| 0 | Reference | | | Reference | | |
| 1 | 1.51 | 1.04-2.18 | 0.0310 | 1.45 | 0.98-2.13 | 0.0609 |
| ≧2 | 2.04 | 1.33-3.13 | 0.0010 | 1.83 | 1.14-2.94 | 0.0124 |
| **mutation** |  | | | | | |
| Exon 19 deletion | Reference | | | Reference | | |
| L858R | 1.17 | 0.85-1.59 | 0.3369 | 1.14 | 0.83-1.57 | 0.4344 |
| **Bone metastasis** |  | | | | | |
| No | Reference | | | Reference | | |
| Yes | 1.41 | 1.03-1.94 | 0.0327 | 1.17 | 0.83-1.66 | 0.3712 |
| **Brain metastasis** |  | | | | | |
| No | Reference | | | Reference | | |
| Yes | 1.31 | 0.93-1.84 | 0.1193 | 1.15 | 0.79-1.66 | 0.4663 |
| **Liver metastasis** |  | | | | | |
| No | Reference | | | Reference | | |
| Yes | 1.70 | 0.98-2.95 | 0.0591 | 1.44 | 0.81-2.57 | 0.2108 |
| **Pleura metastasis** |  | | | | | |
| No | Reference | | |  | | |
| Yes | 1.46 | 1.07-1.99 | 0.0168 |  |  |  |
| **Lung metastasis** |  | | | | | |
| No | Reference | | |  | | |
| Yes | 1.06 | 0.74-1.53 | 0.7490 |  |  |  |
| **Adrenal metastasis** |  | | | | | |
| No | Reference | | | Reference | | |
| Yes | 1.20 | 0.67-2.17 | 0.5428 | 1.14 | 0.62-2.10 | 0.6702 |

PS, performance status

**Supplementary Table 1B. univariate and multivariate analysis of PFS in Afatinib group**

|  | **Univariate analysis** | | | **Multivariable analysis** | | |
| --- | --- | --- | --- | --- | --- | --- |
| **Variable** | HR | 95% Cl | *P-value* | HR | 95% Cl | *P-value* |
| **Gender** |  | | | | | |
| Female | Reference | | | Reference | | |
| Male | 1.03 | 0.60-1.78 | 0.9041 | 1.34 | 0.70-2.57 | 0.3765 |
| **Age** |  | | | | | |
| ≦65 years | Reference | | | Reference | | |
| >65 years | 0.70 | 0.40-1.22 | 0.2079 | 1.30 | 0.66-2.53 | 0.4462 |
| **Smoking status** |  | | | | | |
| Never smoker | Reference | | |  | | |
| Former smoker | 0.77 | 0.40-1.49 | 0.4360 |  |  |  |
| Current smoker | 1.74 | 0.84-3.59 | 0.1345 |  |  |  |
| **Stage** |  | | | | | |
| Recurrence | Reference | | |  | | |
| Ⅲ ^a^ |  |  |  |  |  |  |
| Ⅳ | 2.34 | 1.19-4.60 | 0.014 |  |  |  |
| **PS** |  | | | | | |
| 0 | Reference | | | Reference | | |
| 1 | 2.39 | 1.23-4.67 | 0.0109 | 2.02 | 0.93-4.36 | 0.0737 |
| ≧2 | 2.43 | 1.01-5.83 | 0.0466 | 2.15 | 0.81-5.72 | 0.1267 |
| **mutation** |  | | | | | |
| Exon 19 deletion | Reference | | | Reference | | |
| L858R | 1.06 | 0.54-2.07 | 0.8601 | 1.27 | 0.54-3.01 | 0.5857 |
| **Bone metastasis** |  | | | | | |
| No | Reference | | | Reference | | |
| Yes | 1.38 | 0.79-2.42 | 0.2541 | 1.19 | 0.59-2.42 | 0.6258 |
| **Brain metastasis** |  | | | | | |
| No | Reference | | | Reference | | |
| Yes | 1.49 | 0.84-2.64 | 0.1741 | 1.42 | 0.74-2.73 | 0.2950 |
| **Liver metastasis** |  | | | | | |
| No | Reference | | | Reference | | |
| Yes | 2.52 | 0.77-8.27 | 0.1277 | 2.09 | 0.50-8.71 | 0.3132 |
| **Pleura metastasis** |  | | | | | |
| No | Reference | | |  | | |
| Yes | 1.21 | 0.67-2.19 | 0.5355 |  |  |  |
| **Lung metastasis** |  | | | | | |
| No | Reference | | |  | | |
| Yes | 1.15 | 0.60-2.21 | 0.6733 |  |  |  |
| **Adrenal metastasis** |  | | | | | |
| No | Reference | | | Reference | | |
| Yes | 1.05 | 0.45-2.48 | 0.9089 | 0.85 | 0.30-2.38 | 0.7540 |

^a^ There was no case in the afatinib group.

PS, performance status

**Supplementary Table 2A. Clinical characteristics of 118 patients with brain metastases**

|  |  | **EGFR-TKIs n(%)** | | |  |
| --- | --- | --- | --- | --- | --- |
| **Characteristic** | **total** | **Gefitinib/ Erlotinib** | **Afatinib** | **Osimertinib** | ***P-value*** |
|  | 118 | 50 | 20 | 48 |  |
| **Median Age (Range)** |  | 72.5(30-87) | 65.0(39-77) | 68.5(46-89) | 0.0126 |
| **Gender** |  |  |  |  |  |
| Male | 47 | 19(38.0) | 9(45.0) | 19(39.6) | 0.8632 |
| Female | 71 | 31(62.0) | 11(55.0) | 29(60.4) |  |
| **Smoking status ^a^** |  |  |  |  |  |
| Never | 67 | 28(57.1) | 10(50.0) | 29(61.7) | 0.1062 |
| Former | 32 | 18(36.7) | 5(25.0) | 9(19.2) |  |
| Current | 17 | 3(6.1) | 5(25.0) | 9(19.2) |  |
| **PS** |  |  |  |  |  |
| 0 | 53 | 25(50.0) | 10(50.0) | 18(37.5) | 0.5090 |
| 1 | 37 | 12(24.0) | 7(35.0) | 18(37.5) |  |
| ≧2 | 28 | 13(26.0) | 3(15.0) | 12(25.0) |  |
| **Stage** |  |  |  |  |  |
| Ⅳ | 97 | 44(88.0) | 15(75.0) | 38(79.2) | 0.3395 |
| Recurrence | 21 | 6(12.0) | 5(25.0) | 10(20.8) |  |
| **Mutation status** |  |  |  |  |  |
| Exon 19 deletion | 56 | 21(42.0) | 15(75.0) | 20(41.7) | 0.0256 |
| L858R | 62 | 29(58.0) | 5(25.0) | 28(58.3) |  |
| **Metastasis** |  |  |  |  |  |
| Pleura | 26 | 15(30.0) | 3(15.0) | 8(16.7) | 0.1991 |
| Contralateral lung | 25 | 16(32.0) | 1(5.0) | 8(16.7) | 0.0270 |
| Bone | 59 | 24(48.0) | 8(40.0) | 27(56.3) | 0.4426 |
| Liver | 12 | 3(6.1) | 0(0.0) | 9(18.8) | 0.0290 |
| Adrenal | 10 | 6(12.0) | 1(5.0) | 3(6.3) | 0.4920 |
| **Local control** |  |  |  |  |  |
| Extraction | 10 | 3(6.1) | 3(15.0) | 4(8.5) | 0.4911 |
| Stereotactic radiosurgery | 30 | 16(32.0) | 8(40.0) | 6(12.8) | 0.0239 |
| Whole brain radiotherapy | 27 | 11(22.5) | 6(30.0) | 10(21.3) | 0.7297 |

‡P values were calculated by T-Test, Fisher’s exact test or Chi-square test.

^a^ Information was not available for 2 cases

PS, performance status

**Supplementary Table 2B. Clinical characteristics of 160 patients with bone metastases**

|  |  | **EGFR-TKIs n(%)** | | |  |
| --- | --- | --- | --- | --- | --- |
| **Characteristic** | **total** | **Gefitinib/ Erlotinib** | **Afatinib** | **Osimertinib** | ***P-value*** |
|  | 160 | 68 | 29 | 63 |  |
| **Median Age (Range)** |  | 72(32-78) | 64(32-78) | 70(44-92) | 0.0070 |
| **Gender** |  |  |  |  |  |
| Male | 70 | 26(38.2) | 16(55.2) | 28(44.4) | 0.3027 |
| Female | 90 | 42(61.8) | 13(44.8) | 35(55.6) |  |
| **Smoking status ^a^** |  |  |  |  |  |
| Never | 92 | 39(58.2) | 11(39.3) | 42(70.0) | 0.0449 |
| Former | 47 | 23(34.3) | 11(39.3) | 13(21.7) |  |
| Current | 16 | 5(7.5) | 6(21.4) | 5(8.3) |  |
| **PS** |  |  |  |  |  |
| 0 | 73 | 33(48.5) | 14(48.3) | 26(41.3) | 0.5598 |
| 1 | 53 | 18(26.5) | 10(34.5) | 25(39.7) |  |
| ≧2 | 34 | 17(25.0) | 5(17.2) | 12(19.1) |  |
| **Stage** |  |  |  |  |  |
| Ⅳ | 133 | 56(82.4) | 24(82.8) | 53(84.1) | 0.9623 |
| Recurrence | 27 | 12(17.7) | 5(17.2) | 10(15.9) |  |
| **Mutation status ^b^** |  |  |  |  |  |
| Exon 19 deletion | 84 | 29(43.3) | 27(93.1) | 28(44.4) | <.0001 |
| L858R | 75 | 38(56.7) | 2(6.9) | 35(55.6) |  |
| **Metastasis** |  |  |  |  |  |
| Pleura | 47 | 23(33.8) | 7(24.1) | 17(27.0) | 0.5473 |
| Contralateral lung | 44 | 17(25.0) | 7(24.1) | 20(31.8) | 0.6277 |
| Brain | 59 | 24(35.3) | 8(27.6) | 27(42.9) | 0.3471 |
| Liver | 29 | 12(17.7) | 3(10.3) | 14(22.2) | 0.3856 |
| Adrenal | 18 | 9(13.2) | 4(13.8) | 5(7.9) | 0.5630 |
| **Number of metastatic sites(n)** |  |  |  |  |  |
| One site | 51 | 25(36.8) | 10(34.5) | 16(25.4) | 0.2503 |
| Two sites | 31 | 8(11.8) | 7(24.1) | 16(25.4) |  |
| Three and more sites | 78 | 35(51.5) | 12(41.4) | 31(49.2) |  |
| **Bone modifying agents** |  |  |  |  |  |
| Denosumab | 55 | 30(44.1) | 6(20.7) | 19(30.2) | 0.0560 |
| Zoledronic acid | 43 | 15(22.1) | 12(41.3) | 16(25.4) | 0.1369 |
| **Radiation therapy** | 19 | 20(29.4) | 9(31.0) | 21(33.3) | 0.8892 |

‡P values were calculated by T-Test, Fisher’s exact test or Chi-square test.

^a^ Information was not available for 6 cases

^b^ Information was not available whether it was exon 19del or L858R for 1 case

PS, performance status

**Supplementary Table 2C. Clinical characteristics of 34 patients with liver metastases**

|  |  | **EGFR-TKIs n(%)** | | |  |
| --- | --- | --- | --- | --- | --- |
| **Characteristic** | **total** | **Gefitinib/ Erlotinib** | **Afatinib** | **Osimertinib** | ***P-value*** |
|  | 34 | 14 | 3 | 17 |  |
| **Median Age (Range)** |  | 74(59-78) | 73.5(30-83) | 74(46-84) | 0.7200 |
| **Gender** |  |  |  |  |  |
| Male | 14 | 5(35.7) | 1(33.3) | 8(47.1) | 0.7821 |
| Female | 20 | 9(64.3) | 2(66.7) | 9(52.9) |  |
| **Smoking status** |  |  |  |  |  |
| Never | 17 | 6(42.9) | 3(100.0) | 8(47.1) | 0.3903 |
| Former | 11 | 6(42.9) | 0(0.0) | 5(29.4) |  |
| Current | 6 | 2(14.2) | 0(0.0) | 4(23.5) |  |
| **PS** |  |  |  |  |  |
| 0 | 11 | 7(50.0) | 0(0.0) | 4(23.5) | 0.3891 |
| 1 | 14 | 4(28.6) | 2(66.7) | 8(47.1) |  |
| ≧2 | 9 | 3(21.4) | 1(33.3) | 5(29.4) |  |
| **Stage** |  |  |  |  |  |
| Ⅳ | 28 | 10(71.4) | 3(100.0) | 15(88.2) | 0.3333 |
| Recurrence | 6 | 4(28.6) | 0(0.0) | 2(11.8) |  |
| **Mutation status** |  |  |  |  |  |
| Exon 19 deletion | 18 | 8(57.1) | 3(100.0) | 7(41.2) | 0.1564 |
| L858R | 16 | 6(42.9) | 0(0.0) | 10(58.8) |  |
| **Metastasis** |  |  |  |  |  |
| Pleura | 15 | 6(42.9) | 3(100.0) | 6(35.3) | 0.1138 |
| Contralateral lung | 12 | 4(28.6) | 2(66.7) | 6(35.3) | 0.4561 |
| Brain | 12 | 3(21.4) | 0(0.0) | 9(52.9) | 0.0768 |
| Bone | 29 | 12(85.7) | 3(100.0) | 14(82.4) | 0.7274 |
| Adrenal | 6 | 2(14.2) | 1(33.3) | 3(17.7) | 0.7346 |
| **Number of metastatic sites** |  |  |  |  |  |
| Single | 15 | 6(42.9) | 2(66.7) | 7(41.2) | 0.7093 |
| Multiple | 19 | 8(57.1) | 1(33.3) | 10(58.8) |  |

‡P values were calculated by T-Test, Fisher’s exact test or Chi-square test.

PS, performance status

**Supplementary Table 3. Second-line treatments after Gefitinib/Erlotinib or Afatinib failure**

|  |  | **EGFR-TKIs n(%)** | |  |
| --- | --- | --- | --- | --- |
|  | **Total** | **Gefitinib/ Erlotinib** | **Afatinib** | ***P*-value** |
|  | 238 | 183 | 55 |  |
| **Second-line treatment** |  |  |  |  |
| Osimertinib | 76 (31.9) | 61 (33.3) | 15 (27.3) | 0.3979 |
| Cytotoxic chemotherapy | 162 (68.1) | 122 (66.7) | 40 (72.7) |  |
|  |  |  |  |  |
| **T790M mutation** |  |  |  |  |
| Detected | 68 (28.6) | 55 (30.1) | 13 (23.6) | 0.3555 |
| Undetected or unmeasurable | 170 (71.4) | 128 (69.9) | 42 (76.4) |  |

‡P values were calculated by Chi-square test.

**
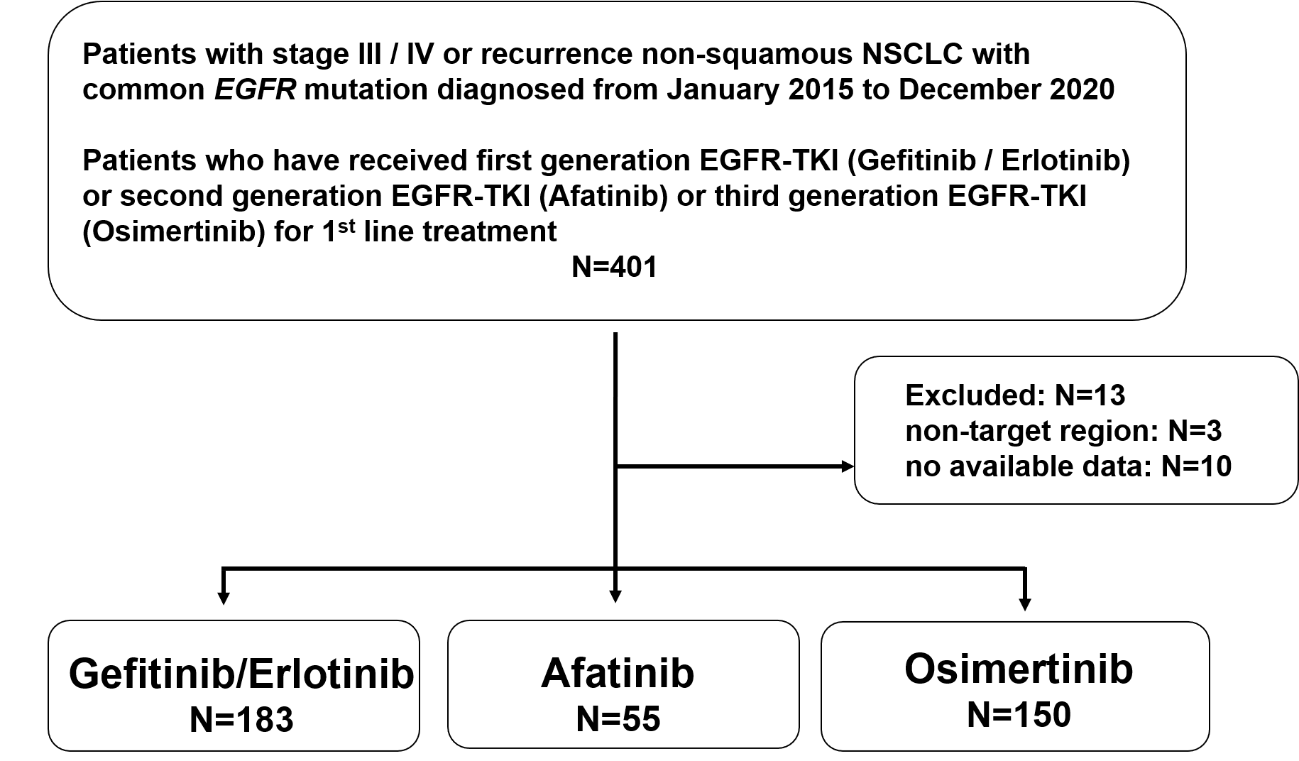
**

**Supplementary Figure 1.**

Flowchart of patient selection in this cohort.


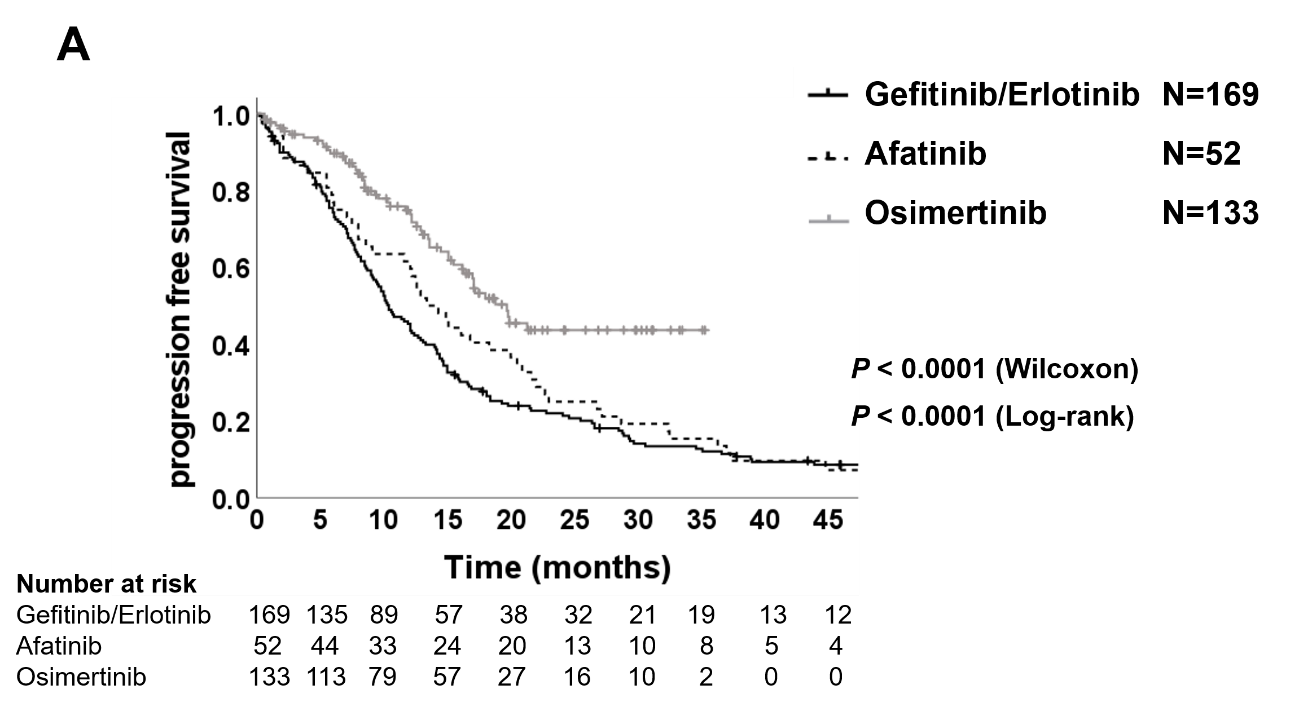

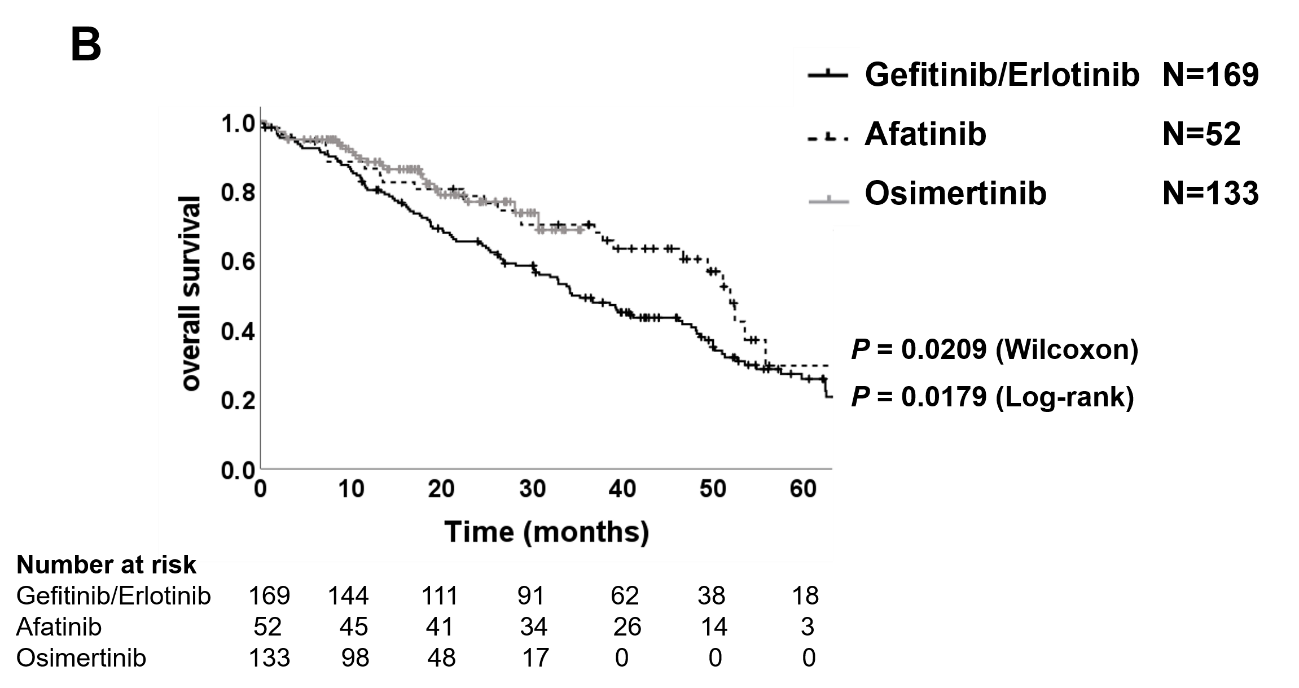


**Supplementary Figure 2.**

Kaplan–Meier plot of progression-free survival (A) and overall survival (B) in patients without liver metastases.


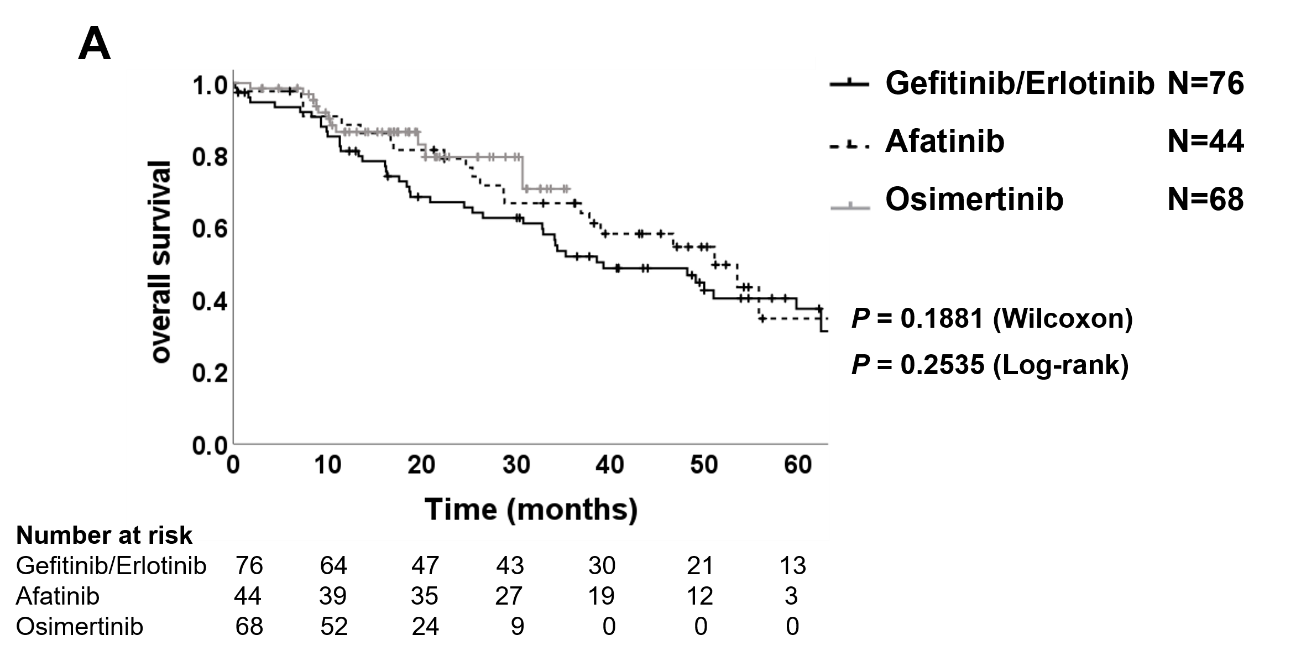

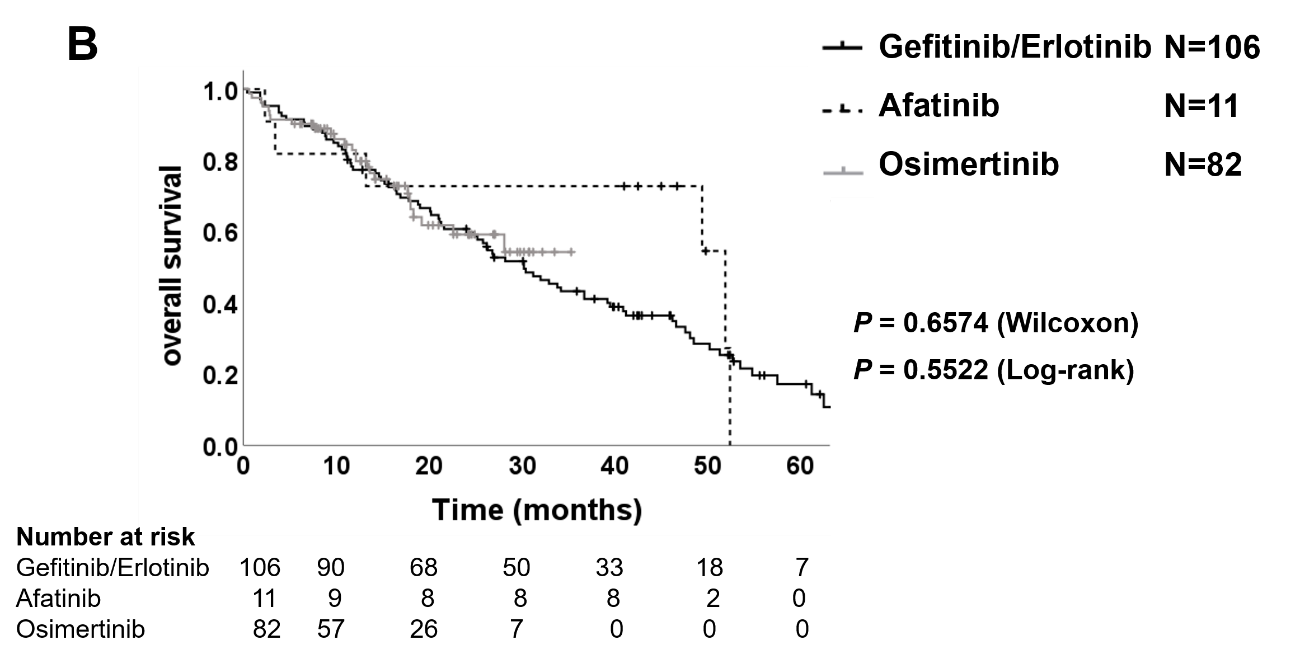


**Supplementary Figure 3.**

Kaplan–Meier plot of overall survival in patients with exon 19 deletion mutation (A) in patients with the L858R mutation (B).


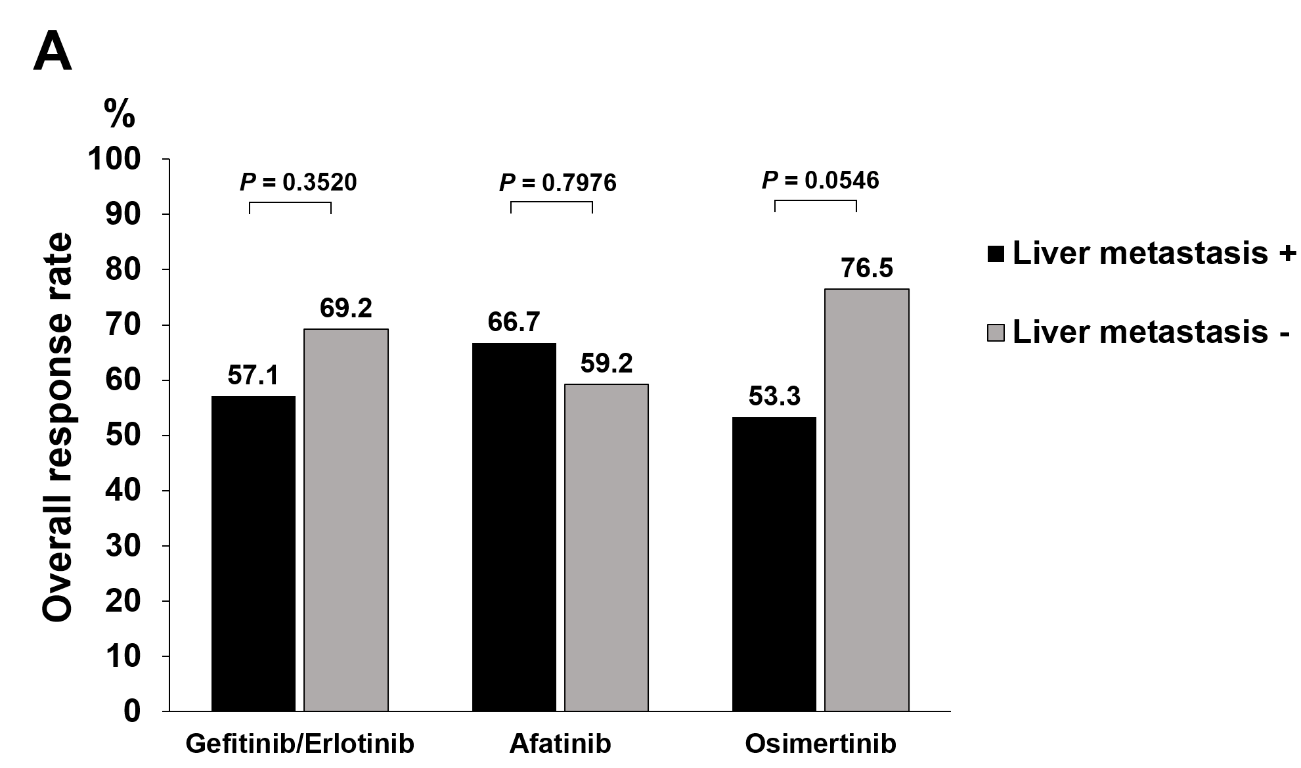

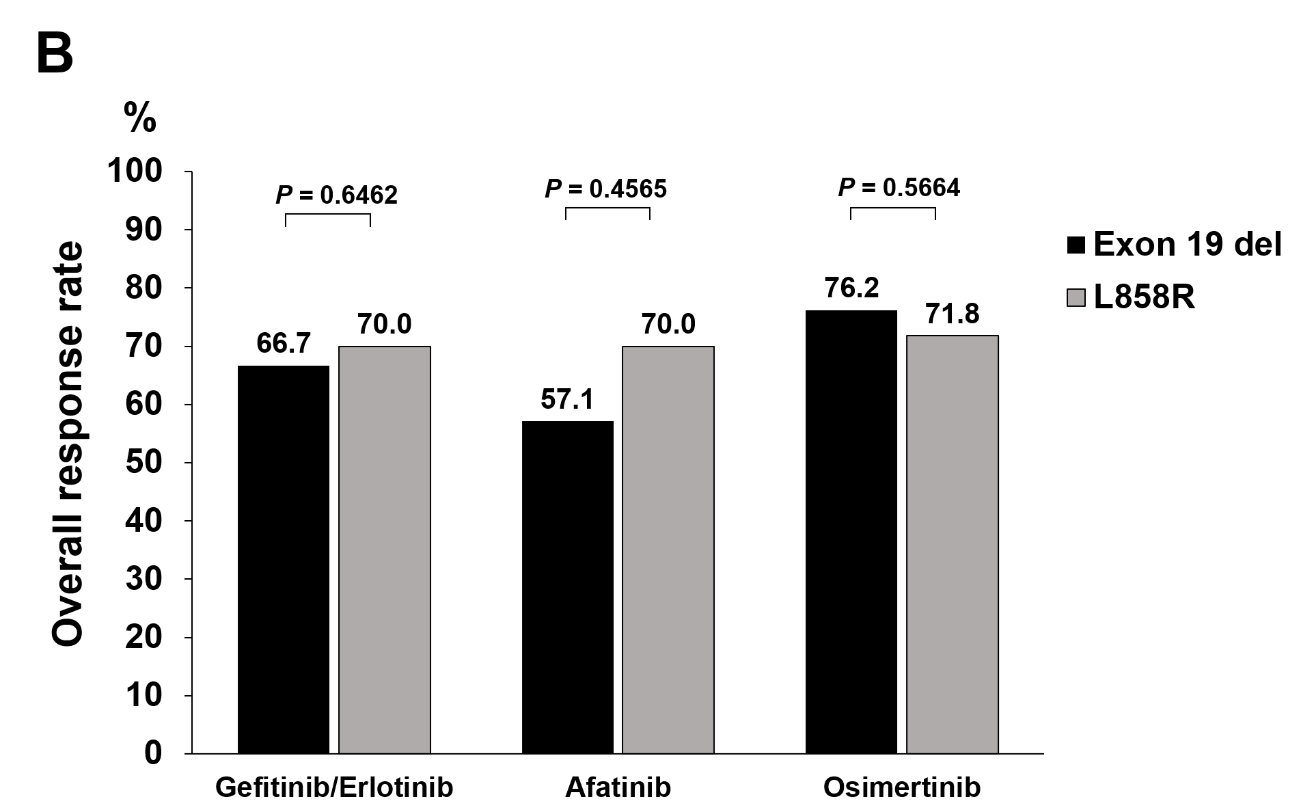


**Supplementary Figure 4.**

Objective response rate in three EGFR-TKI groups with or without liver metastasis (A) and exon 19 deletion or L858R (B). Tumor responses were assessed by the investigators according to the Response Evaluation Criteria in Solid Tumors (RECIST) version 1.1. *P* values were calculated using the chi-square test. Thirteen cases in the gefitinib/erlotinib group, three in the afatinib group, and 16 in the osimertinib group could not be evaluated.
